# An Emergent Role for Mitochondrial Bioenergetics in the Action of Snake Venom Toxins on Cancer Cells

**DOI:** 10.3389/fonc.2022.938749

**Published:** 2022-07-18

**Authors:** Félix A. Urra, Dan E. Vivas-Ruiz, Eladio Flores Sanchez, Ramiro Araya-Maturana

**Affiliations:** ^1^ Laboratorio de Plasticidad Metabólica y Bioenergética, Programa de Farmacología Clínica y Molecular, Instituto de Ciencias Biomédicas, Facultad de Medicina, Universidad de Chile, Santiago, Chile; ^2^ Network for Snake Venom Research and Drug Discovery, Santiago, Chile; ^3^ Interdisciplinary Group on Mitochondrial Targeting and Bioenergetics (MIBI), Talca, Chile; ^4^ Laboratorio de Biología Molecular, Facultad de Ciencias Biológicas, Universidad Nacional Mayor de San Marcos, Ciudad Universitaria, Lima, Peru; ^5^ Laboratory of Biochemistry of Proteins from Animal Venoms, Research and Development Center, Ezequiel Dias Foundation, Belo Horizonte, Brazil; ^6^ Laboratorio de Productos Bioactivos, Instituto de Química de Recursos Naturales, Universidad de Talca, Talca, Chile

**Keywords:** OXPHOS (oxidative phosphorylation), electron transport chain, snake venom, cardiolipin, mitochondrial dysfunction, migrastatics, anticancer compounds

## Abstract

Beyond the role of mitochondria in apoptosis initiation/execution, some mitochondrial adaptations support the metastasis and chemoresistance of cancer cells. This highlights mitochondria as a promising target for new anticancer strategies. Emergent evidence suggests that some snake venom toxins, both proteins with enzymatic and non-enzymatic activities, act on the mitochondrial metabolism of cancer cells, exhibiting unique and novel mechanisms that are not yet fully understood. Currently, six toxin classes (L-amino acid oxidases, thrombin-like enzymes, secreted phospholipases A2, three-finger toxins, cysteine-rich secreted proteins, and snake C-type lectin) that alter the mitochondrial bioenergetics have been described. These toxins act through Complex IV activity inhibition, OXPHOS uncoupling, ROS-mediated permeabilization of inner mitochondrial membrane (IMM), IMM reorganization by cardiolipin interaction, and mitochondrial fragmentation with selective migrastatic and cytotoxic effects on cancer cells. Notably, selective internalization and direct action of snake venom toxins on tumor mitochondria can be mediated by cell surface proteins overexpressed in cancer cells (e.g. nucleolin and heparan sulfate proteoglycans) or facilitated by the elevated Δψm of cancer cells compared to that non-tumor cells. In this latter case, selective mitochondrial accumulation, in a Δψm-dependent manner, of compounds linked to cationic snake peptides may be explored as a new anti-cancer drug delivery system. This review analyzes the effect of snake venom toxins on mitochondrial bioenergetics of cancer cells, whose mechanisms of action may offer the opportunity to develop new anticancer drugs based on toxin scaffolds.

## Introduction

Energy requirements of cancer cells vary significantly from normal cells; they exhibit different metabolic phenotypes with dynamic contributions of both oxidative phosphorylation (OXPHOS) and glycolysis (1) in a process known as metabolic plasticity (2). Notably, a switch to mitochondrial metabolism correlates with metastatic abilities (3, 4) and chemoresistance using alternative energy substrates such as lactate and glutamine (5, 6). Beyond the role of mitochondria in apoptosis initiation/execution, several mitochondrial adaptations support the migration and invasion of cancer cells such as enhanced Complex I activity-dependent reactive oxygen species (ROS) production (3, 4, 7), mitochondrial localization in focal adhesions for local ATP synthesis required for actin cytoskeleton reorganization (8, 9), and high tricarboxylic acid (TCA) cycle activity (10, 11). All these characteristics are associated with more invasiveness and motility (12) and highlight mitochondria as a promising target for metastasis treatment (13).

Snake venoms are a natural source of active proteins, also known as toxins, which exhibit enzymatic or non-enzymatic activities, and are useful molecular scaffolds to develop agents with high affinity and selectivity on cancer cell models *in vitro* and *in vivo* (14–17). Currently, at least nine classes of snake toxins are recognized by suppressing the cancer hallmarks such as ROS-dependent DNA damage, blockage of extracellular matrix-integrin signaling, disruption of cytoskeleton network, inhibition of growth factor-dependent signaling, and induction of OXPHOS dysfunction (13, 18–20). Notably, emergent evidence suggests that several of these toxins act on the mitochondrial metabolism of cancer cells (21–24), exhibiting unique and novel mechanisms that are not yet fully understood. This offers the opportunity to develop new anticancer drugs based on toxin scaffolds. This review analyzes the effect of snake venom toxins on mitochondrial bioenergetics in cancer cells.

### Snake Toxins That Target Mitochondrial Bioenergetics in Cancer Cells

**Figure 1:**
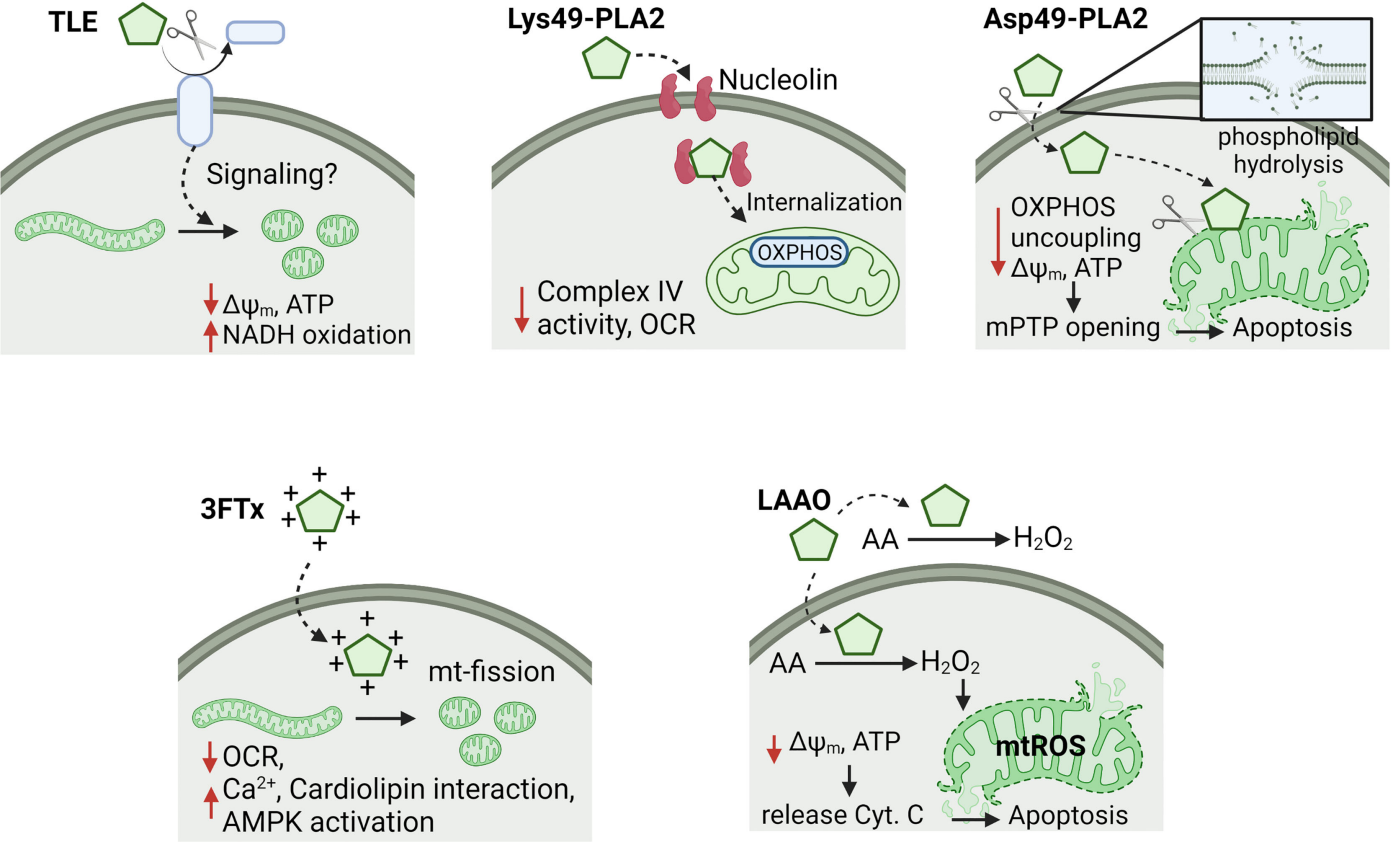
Effects of snake toxins on mitochondrial bioenergetics in cancer cells. It is represented the mechanisms of action of thrombin-like enzymes (TLE), secreted Lys49- and Asp49-phospholipases A2 (PLA2), three-finger toxins (3FTx) and L-amino acid oxidases (LAAO). Some toxins affect the oxidative phosphorylation (OXPHOS) by uncoupling or Complex IV inhibition, which decreases the mitochondrial membrane potential (Δψ_m_) and ATP levels. Moreover, other toxins produce mitochondrial fragmentation (e.g. TLE and 3FTx) or permeabilization of inner mitochondrial membrane (e.g. Asp49-PLA2 and LAAO), triggering apoptosis. Cysteine-rich secreted proteins, and snake C-type lectin toxins were excluded of this figure due to reduced information of a putative mechanism of action on mitochondria. OCR, oxygen consumption rate; Cyt. C, cytochrome C; mPTP, mitochondrial permeability transition pore; mtROS, mitochondrial ROS; AA, amino acids; mt-fission, mitochondrial fission.

Six classes of snake venom toxins with effects on mitochondrial bioenergetics of cancer cells have been studied (**Table 1**). Although the anti-cancer mechanisms of action are not yet fully understood, different determinants may influence the induction of mitochondrial dysfunction such as the site of toxin-cell (e.g. extra or intra-cellular) interaction, physical-chemistry toxin characteristics (e.g. positive charge) to facilitate mitochondrial uptake, and grade of intervention of the mitochondrial metabolism (e.g. permeabilization of the mitochondrial inner membrane, OXPHOS uncoupling, inhibition of respiration, between others). Below, these toxins are discussed in detail (**Figure 1**).

**Table 1:** Toxicological mechanisms of snake venom toxins with effects on mitochondrial bioenergetics of cancer cells.

Name of toxin/snake species	Biochemical characteristics	Mechanism of toxic action	Action on mitochondria
Thrombin-like enzymes (TLE)			
Pictobin Isolated from *Bothrops pictus* venom	49 kDa monomeric serine protease (233 aa) with thombin-like activity and high content of carbohydrates (40%) (25)	Coagulation of plasma and fibrinogen, releasing fibrinopeptide A and induces the formation of a friable/porous fibrin network; also promoted platelet aggregation in human PRP and defibrination in mouse model (23, 25)	Induction of strong NADH oxidation, Δψm, and ATP decrease, triggering mitochondrial fragmentation. Pictobin blocks the fibronectin-stimulated migration in cancer cells (23).
Secreted Phospholipase A2 toxins			
β-bungarotoxin Isolated from *Bungarus multicinctus* venom	23-21 kDa heterodimeric presynaptic neurotoxin with PLA2 subunit (MW: 14 kDa; 120 aa) plus Kunitz-type protease inhibitors subunit (MW: 7 kDa; 60 aa) (26).	Inhibition of neurotransmitter release from presynaptic membranes by depolarizing permeabilization of the synaptosomal plasma membrane (27). Kunitz subunit serves to guide the toxin to its site of action on the presynaptic membrane by virtue of a high-affinity interaction with a specific subclass of voltage-sensitive potassium channels (28).	Possible uncoupling effect of OXPHOS, involving an increase of Ca^2+^-dependent, oligomycin-insensitive respiration, and limiting the state 3ADP respiration (29–33).
Taipoxin Isolated from *Oxyuranus scutellatus scutellatus* venom	45.6 kDa trimeric presynaptic neurotoxin with α subunit (MW: 13,8 kDa; 119 aa), β subunit (β1 and β2 MW: 13.2; 118 aa) and γ subunit (MW: 18.5; 133 aa) (34, 35).	The α subunit phospholipase activity produce fatty acids and lysophospholipids that blockade the neurotransmitter release (36). The neurotoxic effects is due to the formation of bulges along neurites with redistribution of SV proteins within those bulges by bind to pentraxins membrane receptors (NP1, NP2 and NPR) and taipoxin-associated calcium-binding protein 49 (37).	Induction of Δψm drop, generating round and swollen mitochondria, and facilitating the mPTP opening (33).
Ammodytoxin (Atx) Isolated from *Vipera a. ammodytes* venom.	14 kDa monomeric presynaptic β-neurotoxin (38) with anticoagulant effects through its C-terminal and β-wing regions that bins to FXa (39).	Blocking the release of acetylcholine from peripheral neurons at the neuromuscular junction (40). ATx inhibits prothrombinase-complex formation by a non enzymatic, phospholipid-independent mechanism through direct binding to human blood coagulation factor Xa (39).	Binding to subunit II of mitochondrial complex IV, affecting its enzymatic activity (41).
Notexin, Isolated from *Notechis scutatus scutatus* venom.	14 kDa presynaptic neurotoxin (42).	Inhibition of the neuromuscular junction by interfering with presynaptic neurons. It is both neurotoxic and myotoxic, and it causes the degeneration of both muscles and nerves (42)	Induction of Δψm drop and mPTP opening (33).
BaMtx Isolated from *Bothrops atrox* venom.	13 kDa or 24 kDa under reducing or non-reducing conditions, respectively. Monomeric protein with poor phospholipase A2 enzymatic activity (Proleón et al., 2022).	Induction of myotoxicity and production of edema (45).	Mild anti-proliferative and anti-migratory effects on breast cancer cells, affecting the ROS and NADH levels, reducing the mitochondrial respiration (45).
Crotamine Isolated from *Crotalus durissus terrificus* venom	4.8 kDa highly basic (pI = 10.3) cell-penetrating myotoxin (42 aa) (43, 44).	Depolarization and contraction of skeletal muscle *via* interaction with the mammalian voltage Na+ channel, which causes late vacuolization of the sarcoplasmic reticulum and myonecrosis (46). It also selectively inhibits and interferes with the functioning of Kv1.3 channels, promotes the permeability of bacterial membranes (47).	Induction of OXPHOS uncoupling and mitochondrial swelling dependent on Ca^2+^ uptake by mitochondrial calcium uniporter and mPTP opening (48, 49).
Three-finger toxins			
Cardiotoxin VII4 (CTX3) Isolated from *Naja mossambica mossambica* venom	6.7 kDa S-type monomeric β-sheet protein (60 aa) that possesses an unusually high positive charge and invariably serine residue at position 28 of loop II (50, 51).	Strong interaction with anionic phospholipids producing systolic heart arrest by membrane depolarization, cell lysis and transport inhibition (52, 53).	Selectively targeting on mitochondrial membranes probably by binding to cardiolipin, leading to impairs mitochondrial bioenergetics (22).
Cardiotoxin CTX3 Isolated from *Naja atra* venom	6.5 kDa highly basic, hydrophobic, toxic β-sheet protein (60 aa) with pI > 10 (54). It represents the major CTX in the Taiwanese cobra and contains different binding and cytolytic domains (55, 56).	Action on muscular and nervous cells and thereby cause depolarization of excitable membranes associated with binding of cardiotoxin to the cell membrane and disrupting membrane organization and function (54).	Induction of oxidative stress, Δψm decrease, and release of cytochrome c, activating the apoptotic intrinsic pathway and alters mitochondrial biogenesis (57).
L-amino acid oxidases (LAAO)			
ACTX6 and ACTX8 Isolated from *Agkistrodon acutus* venom	ACTX8: 28 kDa cysteine-rich single chain protein containing four disulfide bonds (pI = 8.2) ACTX6: 97 kDa homodimer protein.	ACTX-8 and ACTX6 oxidizes L-type amino acids to produce H_2_O_2_ and ammonia. The oxidation reaction can generate oxidative stress on cells and induce cell apoptosis (58, 59).	Induction of Δψm drop, inducing translocation of cytochrome c to cytosol, initiating the intrinsic apoptosis pathway (58, 59).
Rusvinoxidase Isolated from *Daboia russelii russelii* venom	57 kDa acidic monomeric glycoprotein with yellow coloration due to presence of flavin adenine dinucleotide (60).	No biological activity in envenoming is reported. But induces apoptosis by both the extrinsic (death-receptor) and intrinsic (mitochondrial) signaling pathway in cancer cell (60, 61).	Induction Δψm drop, inducing translocation of cytochrome c to cytosol, initiating the intrinsic apoptosis pathway (60, 61).
Cysteine-rich secreted proteins (CRISP)			
Natrin Isolated from *Naja naja atra* venom	25 kDa toxin protein (221 aa) with tree regions: N-terminal pathogenesis-related protein of the group 1 (PR-1) domain, a C-terminal cysteine-rich domain (CRD), and a hinge region linking the two motifs (62).	It produces high-conductance calcium-activated potassium (BKCa) channel, inhibition of Kv1.3 (63), inhibition of ryanodine receptors (64), and inflammatory modulation (65).	Induction of rewiring of mitochondria-participating metabolic pathways such as sphingolipid/glycerophospholipid metabolism, FA biosynthesis, and oxidation (66).

### Thrombin-Like Enzymes

Thrombin-like enzymes (TLEs) are serine proteases belonging to family S1 (chymotrypsin), subfamily A of the trypsin-like group (67). Its active site (His57, Asp102, Ser195) catalytically releases fibrinopeptide A or B from Aα or Bβ fibrinogen’s chain cleavage, respectively, but rarely both fibrinopeptides, as thrombin (68). The molecular mass of TLEs varies between 26 and 67 kDa, depending on the content of its carbohydrates (N- or O-linked) (69, 70). The tertiary structure shows a β/β hydrolase type fold with variants in the shape of certain regions (mainly loops) that attribute particular functional mechanisms (71). TLEs have only up to 33% homology with thrombin and differ with it in 1) preference for fibrinogen chain cleavage; 2) absence of allosteric modulators; 3) insensitivity to antithrombin III, hirudin, and heparin, and 4) do not activate another coagulation factor (72). TLEs produce a coagulating effect *in vitro*; however, *in vivo*, form atypical fibrin meshes that are quickly degraded by secondary fibrinogenolytic processes, therefore, endogenous fibrinogen decreases considerably, making the blood uncoagulable (73). Additionally, TLEs produce alterations in the nervous system (74), complement system (75), muscle (76), and potassium channel blocking (77).

Pictobin is a 41-kDa glycoproteinase that coagulates plasma and fibrinogen, releasing fibrinopeptide A and promoting the formation of a friable/porous fibrin network (23). Platelets and cancer cells can interact between them or with extracellular cues to modify their reactivity or metastatic ability, respectively (13, 78). In this context, there is experimental evidence that pictobin triggers mitochondrial dysfunction in cancer cells. This toxin inhibits the fibronectin-dependent migration in lung and breast cancer cells and produces strong NADH oxidation, Δψm depolarization, ATP decrease and fragmentation of the mitochondrial network at non-cytotoxic concentrations (23). Currently, the molecular mechanism of mitochondrial dysfunction remains unknown; however, a probable intracellular signaling initiated in the plasma membrane by cleavage of some receptor in cancer cells (**Figure 1**), like to thrombin in platelets, may be occurring (79, 80). This may represent a novel mechanism of new anti-metastatic approaches by interfering with extracellular cues-metabolism communication (13).

### Secreted Phospholipase A2 Toxins

Secreted phospholipases A2 present in some snake venoms (svPLA2s) are Ca^+2^-dependent enzymes that catalyze the hydrolysis of phospholipids at *sn-2* positions, producing lysophospholipids and free fatty acids. Structurally, these toxins have three major α-helices and two antiparallel β-sheets linked by disulfide bonds. The residues His48, Asp49, Tyr52, and Asp99 make up the active site, coordinating Ca^2+^ by a hydrogen bond (81). Remarkably, svPLA2 group IIA is subdivided into two types according to their evolutionary pathway: classical PLA2s and PLA2 homologs (or PLA2-like proteins). The latter group exhibits substitutions in the catalytic domain (Asp49Lys) and the calcium-binding loop (Tyr28Asn) (82, 83), lacking phospholipase activity. Usually, they are also referred to as Lys49-PLA2s (K49-PLA2s). Notably, this svPLA2 diversity correlates with several biological actions, such as presynaptic/postsynaptic neurotoxicity, myotoxicity, cardiotoxicity (84), platelet aggregation inhibition, and edema-inducing effects (85). Moreover, antiviral effects against Dengue virus (86), antibacterial (87), antiparasitic 21), and antitumoral (88) activities have been also reported for svPLA2. A selected svPLA2 set interferes with mitochondrial bioenergetics, affecting the migration, proliferation, and viability of cancer cells, as is discussed below.

In human neuroblastoma SK-N-SH cells, notexin produces an increase in intracellular Ca^2+^ and ROS levels, activating p38 MAPK/ATF-2 and JNK/c-Jun signaling pathways. These, in turn, trigger the modulating expression of Bcl-2 family proteins, which produces Δψm drop, mitochondrial permeability transition pore (mPTP) opening, and consequently, intrinsic apoptosis pathway (89).

Taipoxin, notexin, and β-bungarotoxin selectively affect the mitochondria of nerve terminals (32), lacking the effects on Ca^2+^ transport into liver mitochondria (90). These neurotoxins induce the Ca^2+^ accumulation within nerve terminals, and bind the mitochondria, producing Δψm drop, generating round and swollen mitochondria, and facilitating the mPTP opening (33). Interestingly, lysophospholipid-fatty acid (FA) mixtures (two products generated by PLA2 activity) recapitulate the mitochondrial effects of these neurotoxins, suggesting a relevant role of local lipid changes in synaptic vesicle release during paralysis of the neuromuscular junction (91). Early studies on the effect of β-bungarotoxin on mitochondrial bioenergetics have suggested an uncoupling effect of OXPHOS (29–31, 90), involving an increase of Ca^2+^-dependent, oligomycin-insensitive respiration in synaptosomes (31). The FA released by PLA2 action at the plasma membrane, which diffuses across the cytosol and interacts with the mitochondrial inner membrane, apparently is a mechanism involved in this OXPHOS uncoupling (31). Moreover, a toxin-induced limitation in the maximal rate of electron transport (in presence of oxidable substrates such as pyruvate, glutamate, succinate, and TMPD plus ascorbate) can be observed in toxin-treated tissues, limiting the state 3ADP respiration (30). Notably, β-bungarotoxin is also a known inhibitor of α7 nicotinic acetylcholine receptors (α7nAChR) (92, 93); however, if inhibition of these receptors is involved in the observed effect on the mitochondrial bioenergetics remains currently elusive. In non-small cell lung carcinomas (NSCLC) cells under nicotine stimulation, β-bungarotoxin reduces the proliferation *in vitro* and tumor growth *in vivo* (94). Moreover, β-bungarotoxin reduces the invasion *in vitro* of primary cultures of cancer cells derived from 27 NSCLC tissue samples (95).

Ammodytoxin (Atx), a β-neurotoxin, is uptake intracellularly during a retrograde transport from the cell surface by binding to protein disulfide isomerase (PDI). The Atx-binding site on human PDI is located on an extensive area on its interfacial binding surface between domains b and b´ like the known binding sites of endogenous PLA2 such as the mammalian GIB, GIIA, GV, and GX PLA2s (96). In neuron cytosol, Atx binds to calmodulin and 14-3-3 proteins (97–99), which may be involved in the passage of Atx from the cytosol to nucleus and mitochondria (100). For decades R25, a 25KDa-putative mitochondrial protein, was considered as the receptor for Atx in neuronal mitochondria (101, 102). Recently, the R25 identity was revealed as a part of the subunit II of cytochrome c oxidoreductase, which is encoded by the mitochondrial *MT-CO2* gene (41). In rat adrenal pheochromocytoma cells, Atx and its catalytically inactive mutant Atx(D49S) colocalize with mitochondria at 5 min of incubation and inhibit the Complex IV activity, suggesting that the PLA2 activity is dispensable for the Atx-Complex IV interaction 41). Remarkably, the subunit II of Complex IV is positioned to face the mitochondrial intermembrane space, being accessible to Atx arriving from the cytosol. The effects on mitochondrial bioenergetics and metabolic signaling pathways triggered by Complex IV inhibition by Atx still remain unknown.

Crotamine, a PLA2 with positively-charged 42 amino acids, 24) presents both cationic and hydrophobic regions that allow the interaction with negatively charged phospholipids (44). It is a cell-penetrating myotoxin that enters tumor cells by binding to cell surface heparan sulfate proteoglycans (HSPGs), triggering its endocytosis (103). This characteristic confers it the ability to cross lipid bilayers of cellular membranes and transport cargo into cells. Elegant examples of crotamine as a nanocarrier for non-viral delivery of DNA have been reported (103–105). In cancer cells, crotamine produces a rapid increase of the intracellular Ca^2+^ from the endoplasmic reticulum (ER) and lysosomes, Δψm depolarization, and *in vivo*, it induces selective penetration in proliferating cancer cells and consequently, apoptosis without toxic effect on non-tumoral tissues (48). On the other hand, crotamine promotes OXPHOS uncoupling and mitochondrial swelling dependent on Ca^2+^ uptake by mitochondrial calcium uniporter and mPTP opening (49). Since ER-mitochondria Ca^2+^ transfer is essential for cancer cell bioenergetics (106), metastasis (107), and chemoresistance (108), the anti-cancer effects of crotamine may rely on the disruption of ER-mitochondria axis and activation of Ca^2+^-dependent cytosolic proteases.

BaMtx is a 13 kDa Lys49-PLA2 homologue with high myotoxic activity 45). This effect is attributed to cationic residues in the conserved C-terminal region (109), which electrostatically interacts with negatively charged residues in the cellular membrane collapsing the membrane organization (110). In another Lys49-PLA2 toxins, this favors the penetration and disorganizes bilayers, leading to a Ca^2+^ influx that promotes selective necrosis of myotubes (111). This mechanism could be the basis for the mild anti-proliferative and anti-migratory effects showed BaMtx on breast cancer cells. A possible mechanism dependent on mitochondrial bioenergetics was recently hypothesized (45). BaMtx may increase a fast Ca^2+^ influx, producing oxidative stress that reduces the electron donor NADH for Complex I activity, reducing basal mitochondrial respiration and consequently the precursors required for G1/S-cell cycle transition as have been described for proliferating cancer cells (112, 113). For Lys49-PLA2, cellular uptake mediated by nucleolin is an essential step for the biological effect in several cell lines. Nucleolin is a protein located in the nucleolus, cytoplasm, and on the cell membrane, participating in DNA and RNA metabolism, ribosome biogenesis, and cytokinesis (114). Interestingly, nucleolin is overexpressed and partially localized on the cell surface of cancer cells (115), becoming it a preferential target for the delivery of anticancer agents that target surface nucleolin for potential effective and nontoxic cancer therapy. Mt-II, a Lys49-PLA2 isolated from *Bothrops asper*, interacts with the central RRM and the C-terminal R/F-GG domains of nucleolin at the cell surface and consequently occurring the internalization (116). This mechanism may explain the selective effect of PLA2s on tumor mitochondria and highlight their potential anticancer as Proleón et al. (45) have reported (**Figure 1**).

### Three-Finger Toxins

Snake venom three-finger toxins (3FTxs) are present in the venoms of elapids (cobras, kraits, and mambas), hydrophiids (sea snakes), and some colubrids (117). The 3FTxs are characterized by three finger-like loops rich in β-strands and emerging from a dense, globular core reticulated by four highly conserved disulfide bridges (118). Commonly, they produce neuromuscular junction blocking by inhibition of nicotinic and muscarinic acetylcholine receptors or inhibition of enzyme acetylcholinesterase (119); however, other targets have been described for 3FTxs such as mitochondria in cancer cells.

Basic three-fingered S-type cardiotoxin from *Naja mossambica mossambica* venom translocates to mitochondria to promote apoptosis by inducing mitochondrial dysfunction in neuroblastoma cells (22). This toxin has a high net positive charge and produces fragmentation of the mitochondrial network, mitochondrial mass reduction, a decrease of the basal mitochondrial respiration and spare capacity, promoting mitochondrial permeabilization by electrostatic interaction with cardiolipin, the main phospholipid of the inner mitochondrial membrane (22). Another cardiotoxin, CTX3 from *Naja atra*, targets mitochondria to induce oxidative stress, Δψm decrease, and release of cytochrome c, activating the intrinsic apoptosis pathway (57). Recently, it has been described that CTX3 induces activation of the main sensor of cellular energy status, AMP-activated protein kinase (AMPK) (120, 121), which has an essential role in mitochondrial and lysosomal biogenesis (122–124). CTX3-induced mitochondrial dysfunction produces an increase of intracellular Ca^2+^, triggering autophagy and apoptosis by a Ca^2+^/protein phosphatase 2A (PP2A)/AMPK axis in leukemia cells (121).

### L-Amino Acid Oxidases

L-amino acid oxidases (LAAOs) are homodimeric flavoenzymes with covalently linked-flavin adenine dinucleotides (FADs) that catalyze the oxidative deamination of L-amino acids to α-keto acids, producing ammonia and locally an excess of hydrogen peroxide (H_2_O_2_) in the vicinity of cells triggering oxidative stress and apoptosis (125, 126). LAAOs are present in the venom of Viperidae and Elapidae snake species and exhibit antimicrobial effects (127) and inhibition and activation of platelet aggregation (128–130). In cancer cells, LAAOs produce cell death through H_2_O_2_ accumulation-induced oxidative stress, which causes damage to DNA and cell membranes (131). Although the cytotoxic mechanisms are not fully understood, extensive studies have shown that LAAOs (e.g. Actx6, Actx8, and MipLAAO), decrease Δψm, inducing translocation of cytochrome c to cytosol, initiating the intrinsic apoptosis pathway (58, 60, 132, 133). Interestingly, rusvinoxidase purified from the venom of *Daboia russelii russelii* is a LAAO that induces apoptosis in MCF7 breast cancer cell line in a manner independent of its enzymatic activity. It produces Δψm drop accompanied by an increased ROS production, glutathione depletion, and catalase activity, producing release of cytochrome c, increase of Bak pro-apoptotic protein, and decrease anti-apoptotic proteins Bcl-XL and heat-shock proteins (HSP-90 and HSP-70) (60). Cancer cells with high catalase activity are resistant to toxin-induced death (61). This evidence suggests that intracellular ROS production is an essential step for triggering mitochondria-dependent apoptosis. *In vivo* experiments, rusvinoxidase is non-toxic in mice, indicating that it may be useful as a model for the development of peptide-based anticancer drugs.

### Cysteine-Rich Secreted Proteins

The Cysteine-rich secreted proteins (CRISP) are proteins highly conserved that exhibit a common secondary structure that includes 16 conserved cysteine residues (134). In mammalians, CRISPs are associated with reproduction, cancer, and immune responses. They are also present in venomous glands of non-mammalian animals (e.g. snakes, spiders, scorpions, lizards, and cone snails) (134). Some snake venom CRISP (svCRISP) toxins inhibit depolarization-induced contraction in the arterial smooth muscle of rat tail, interacting with several ion channels, such as L-type Ca^2+^ channels (135), voltage-gated K^+^ channels, and ryanodine receptors (62, 64, 136). The biological effects of svCRISPs involve pro-inflammation and recruiting of neutrophils *in vivo* (65, 137), myotoxicity (138), antimicrobial activity (139), and increased trans-epithelial permeability (140). Natrin, a svCRISP of 221 amino acids and a molecular mass of 25-kDa, induces changes in metabolites such as phytosphingosine, 3-O-sulfogalactosylceramide, ganglioside, glycerophospholipids such as PC and PE, FAs such as lauric and palmitic acids (66). Although the natrin effect on cellular metabolism is not fully understood, these findings suggest that the natrin-induced apoptosis on cancer cells involves a rewiring of mitochondria-participating metabolic pathways such as sphingolipid/glycerophospholipid metabolism, FA biosynthesis, and oxidation (66). Future studies could explore the therapeutic potential of this metabolic phenotype induced by natrin in cancer cells.

### Snake C-Type Lectin

C-type lectins are proteins that recognize and bind to specific carbohydrate domains on the cell membrane, and they are grouped into classical Ca^2+^- and sugar-binding lectins and the non-sugar-binding snake venom C-type lectin-related proteins (SV-CLRPs). These toxins target different coagulation factors and platelet receptors that participate in hemostasis, thrombus formation, inflammation, and metastasis with promising biomedical projections (141). Several cancer cell types under metastatic dissemination interact with platelets *via* podoplanin-C-type lectin-like receptor 2 (CLEC-2). CLEC-2 is a transmembrane receptor that upon activation, homodimerizes and initiates Syk-dependent signaling of collagen receptor GPVI, platelet integrin αIIbβ3, promoting platelet aggregation and thrombus formation (142–144). Interestingly, some C-type lectin from snake venom such as rhodocytin (also termed aggretin) purified from the venom of *Calloselasma rhodostoma* bind to CTLD domain of CLEC-2. This binding is favored by four arginine residues on its lateral face of CLEC-2 (145) and reduces the metastasis promoted by platelet-cancer cell interactions (146). Similarly, the rhodocytin α-chain C-terminus inhibits the platelet aggregation and metastatic abilities of hepatocarcinoma cells (147). Although mitochondrial metabolism is relevant in platelet aggregation (148), the effect of snake C-type lectins on platelet bioenergetics is currently unknown. Remarkably, some lights on the potential effect of snake C-type lectins on mitochondria of cancer cells can be found in the action of BlL toxin. BlL is a dimeric galactose-binding C-type lectin purified from *Bothrops leucurus* snake venom (149) with antibacterial and anticancer effects (150). It induces dose-dependent necrotic cell death in B16-F10 melanoma cells, without affecting the viability of keratinocytes (151). BlL increases the intracellular Ca^2+^ levels, and mitochondrial superoxide, and decreases Δψm, suggesting the induction of cyclosporine A-sensitive Ca^2+^-induced mPTP opening in cancer cells (150, 151).

### Future Perspective and Conclusion

Emergent evidence suggests that several snake venom active components/toxins target the mitochondrial bioenergetics with selective migrastatic and cytotoxic effects on cancer cells. Since some of them additionally disturb platelet function, these toxins also offer novel opportunities for promising lead compounds that reduce platelet-cancer cell interaction and venous thromboembolism often seen observed in cancer. The site of toxin-cell interaction, the physical-chemistry characteristics of toxins, and the type of action on mitochondria (e.g. permeabilization of inner mitochondrial membrane, respiratory complex inhibition, cardiolipin interaction, and uncoupling of OXPHOS) are determinants for anticancer effects. Since cancer cells can exhibit elevated Δψm compared with non-tumor cells, selective accumulation of compounds linked to cationic snake peptides within mitochondria in a Δψm-dependent manner may be explored for a new anti-cancer drug delivery system. Despite the above, evaluation of efficacy, potency and safety are challenges that will need to be established for future *in vivo* applications.

## Author Contributions

FU designed and outlined the structure and contents of the review. FU, DV-R, EF, and RA-M contributed to the literature review, discussion, and writing of the manuscript. All the authors contributed equally to the draft revisions and final approval of the version to be published.

## Funding

This work was funded by Agencia Nacional de Investigación y Desarrollo (ANID-Chile) FONDECYT grants #1221874 (RA-M), #11201322 (FU), VID-University of Chile #UI-024/20 (FU), International collaboration Project ANID #Redbio0027 (FU, RA-M, DV-R, ES), Anillo grant ACT210097 (RA-M, FU), Fondo Nacional de Desarrollo Científico, Tecnológico y de Innovación Tecnológica (FONDECYT-Perú) #079-2021-FONDECYT (DV-R), Fundação de Amparo a Pesquisa do Estado de Minas Gerais (FAPEMIG-Brazil), Grant #APQ-01724-18 (ES), Conselho Nacional de Desenvolvimento Científico e Tecnológico (CNPq-Brazil), and Grant #309823/2021-8 (ES).

## Conflict of Interest

The authors declare that the research was conducted in the absence of any commercial or financial relationships that could be construed as a potential conflict of interest.

## Publisher’s Note

All claims expressed in this article are solely those of the authors and do not necessarily represent those of their affiliated organizations, or those of the publisher, the editors and the reviewers. Any product that may be evaluated in this article, or claim that may be made by its manufacturer, is not guaranteed or endorsed by the publisher.
